# Immunotherapy for Dogs: Running Behind Humans

**DOI:** 10.3389/fimmu.2018.00133

**Published:** 2018-02-05

**Authors:** Hans Klingemann

**Affiliations:** ^1^NantKwest, Inc., Culver City, CA, United States

**Keywords:** comparative oncology, immunotherapy of cancer, canine, veterinary medicine, cancer immunotherapy

## Abstract

A number of excellent reviews on the potential of canine cancer immunotherapy are available, but many extrapolate from observations in humans when in fact only very few immunotherapies have been developed for canines that have shown efficacy in well-designed studies. Pharmaceutical and biotech companies are aware that the market for more expensive immunotherapies in canines is limited resulting in limited funding for clinical trials. However, dogs and other pets deserve access to this new form of cancer therapy. The purpose of this brief review is to summarize the current status of available immunotherapies for dogs and their near-term prospects, provided we can effectively translate discoveries and progress in humans to canines.

## Introduction

Immunotherapy is the new buzzword in human oncology. After decades of weakening or even eliminating the patient’s immune system with chemotherapy, the trend is now to protect and boost that very same immune system [reviewed in Ref. (1)]. The most successful human immunotherapies to date include monoclonal antibodies (mAbs) against lymphoma antigens (i.e., CD20 – rituximab) as well as mAbs against checkpoint molecules such as PD-1 and CTLA-1. Blocking those pathways unleashes the cytotoxic power of T lymphocytes and may also activate other immune responses such as antigen presentation and cytokine release [reviewed in Ref. (2)]. However, antibodies that target checkpoint molecules are not the only class of immunotherapeutics where canine medicine is “running behind humans.” Other examples include tumor-specific cytotoxic immune cells and cytokines. Immunotherapy is not expected to become the panacea for all canine tumors – we know from human patients that some cancers (such as melanoma and lymphoma) respond well to immunotherapy, but solid tumors still have poor responses (3). It is also important to remember that immunotherapy works best with less tumor load and “classical” anti-tumor treatments such as surgery, radiation, and chemotherapy are necessary to reduce tumor load.

Each year, about 6 million of 70 million dogs in the USA alone will develop cancer (4, 5). Why then are efforts to develop immunotherapies for dogs so limited? We still prefer to rely heavily on chemotherapy that affects the general condition of the dog for months and often has limited benefit, with disease recurrence usually within a year. There are several reasons for the limited interest especially by biotech/pharmaceutical industry to develop effective immunotherapies for canines and its challenges (Table 1).

**Table 1:** Challenges in cancer immunotherapy for dogs.

Costs for clinical trials Profit margin for drug/biotech companies limited – less incentive of investing in clinical trials Randomized trials are challenging: Breed variability Heterogeneous tumor biology Rigor in disease staging Outcome measurements require close follow-up Immunotherapy can cause initial tumor enlargement – leading to early discontinuation At progression, owners often prefer euthanasia Owners may consider treatment interventions palliative, often resulting in reduced drug dosing Limited knowledge about canine immune system (lack of reagents or biological markers) Non-invasive detection and predictive response tests lacking (example “liquid biopsy”) Genome guided treatment design still at an early stage of development

First, to conduct well-controlled clinical trials in dogs is challenging considering the breed specifics and costs for clinical trials. Drug and biotech companies are aware that the market for more expensive immunotherapies is limited, and the pharmaceutical industry expects profit margins to be unpredictable but generally less than for human medicines. Conventional cancer treatments with chemotherapy (especially generic drugs) and radiation will usually amount to a few thousand dollars, which however will barely pay for one infusion with a checkpoint inhibitor, a treatment that can run up six figure costs in humans.

Importantly, the canine immune system has not been very well researched and characterized compared to the human immune system. We know relatively little, for example, about lymphocyte subtypes and expression and regulation of their receptors, or about canine cytokines that support their function. In part, this lack of information is due to the limited availability of canine-specific reagents to characterize immune system components. This also implies that there are no predictive tests, which dog may benefit from what type of immunotherapy. Moreover, tumors in dogs have not as well characterized with respect to genotype and phenotype. These limitations hamper efforts to develop mAbs or other targeted therapies for dogs such as chimeric antigen receptor (CAR) cells, in which immune cells are engineered to recognize and respond to a specific antigen on cancer cells.

The National Cancer Institute (NCI) in 2003 initiated the *Comparative Oncology Program* with the goal to foster studies in canines that could inform the designs of trials in humans (4–6). Relatively few studies were completed largely due to limited funding by drug companies that did not necessarily recognize that cancer in human and dogs share significant similarities. Realizing the recent discoveries in immuno-oncology and the fact that studies in dogs can be highly informative for humans, the NCI recently released more significant grant funding to study canine tumor biology, its genetics and immunotherapies.

Canine cancer is responsive to immunotherapy, as first shown by bone marrow transplants between littermates, pioneered in the 1960s and 1970s at the Fred Hutchinson Cancer Research Center in Seattle [reviewed in Ref. (7)]. Those studies received little attention, as their focus was to show that high, myeloablative doses of chemotherapy and radiation can eliminate cancer (mostly leukemia and lymphoma). However, after analysis of larger patient numbers, it became clear that patients who had received an allogeneic transplant had significantly less tumor recurrences than when the patient’s own (autologous) bone marrow was used (8), This beneficial effect was later defined as “*graft versus leukemia*.” largely mediated through allogeneic activated T lymphocytes.

Some decades ago, investigators from the University of Pennsylvania infused cytotoxic T cells from the human T-cell line TALL-104 into dogs with sarcomas, lymphoma, or mammary cancer (9–11). These studies are noteworthy as the TALL-104 clonal cell line was generated from a human patient with leukemia. Despite its allogeneic and even xenogeneic nature, the cells that were irradiated before infusion did not cause adverse events in the recipient dogs, including no tumor formation. In fact, some of the treated dogs achieved a partial response and survival was improved, but TALL-104 cytotherapy never caught on. The cells were too difficult (and too expensive) to maintain in culture and could not be cryopreserved to provide an “off the shelf” treatment.

## Currently Available Immunotherapies for Canine Cancers

What do we currently know about the efficacy of the different types of immunotherapeutic interventions in dogs? Rather than being exhaustive and discuss each canine immunotherapy available or in development, this review aims to point to the challenges and potential solutions. There are a number of review papers that present the spectrum of canine immunotherapies comprehensively (12–15) and a website can guide to currently open trials (https://ebusiness.avma.org/aahsd/study_search.aspx).

### Monoclonal Antibodies

Compared to the plethora of mAbs available for humans, the development of mAbs for dogs lags behind substantially, largely because few targetable tumor antigens have been identified in dogs. At this point, only mAbs for CD20-positive B cell (Blontress^®^) or CD52 positive T cell lymphoma (Tactress^®^) are approved by the US Department of Agriculture and commercially available in the USA and Canada (12). However, the anti-lymphoma efficacy of these mAbs is substantially inferior to what is seen in human patients. Although some human antibodies seem to cross-react with canine epitopes, for example, antibodies to HER-2 for osteosarcoma (16) and CSPG4 for melanoma (17), a single amino acid sequence in a critical position can prevent cross-species reactivity as documented in the case of rituximab.

The example of the different efficacy spectrum of the canine versus the human CD20 mAb raises the questions whether this could be a more general phenomenon. The major mechanism of action for most human mAbs is through antibody-dependent cellular cytotoxicity (ADCC), exclusively mediated through the Fc receptor (FcR) on natural killer (NK) cells (18). Treatment outcomes with mAbs are far superior in human patients if their NK cells express a high-affinity FcR (19, 20). Since we know relatively little about the FcR distribution on canine immune cells, especially on NK cells, optimizing mAb efficacy for dogs is difficult (21). This may explain the suboptimal response to canine CD20 mAb compared to the responses seen in humans. Additional canine CD20 mAbs are under development, and preliminary studies have shown depletion of peripheral B lymphocytes in beagle dogs (22).

As in humans, T-cell lymphoma carries a worse prognosis than B-cell lymphoma. A caninized mAb against the T-cell antigen CD52 (Tactress^®^) has been tested in two large, well-controlled studies in conjunction with cytotoxic chemotherapy for the treatment of canine T-cell lymphoma. Unfortunately, the mAb did not improve progression-free survival in this more aggressive form of lymphoma (23).

Bevacizumab is a humanized mAb that inhibits angiogenesis by blocking the vascular endothelial growth factor (VEGF). In humans, it is used to treat a variety of cancers. It has shown some efficacy in murine models of *canine* mesenchymal neoplasms (hemangiopericytoma and osteosarcoma) (24). The results in mice suggest that some mAbs can have cross-species reactivity, which may vary with the type of epitope recognized by the mAb. A placebo-controlled trial to test the safety and efficacy of bevacizumab for canine hemangiosarcoma is ongoing (25).

The mAbs against the checkpoint molecules CTLA-4 and PD-1 produce remarkable responses in humans, especially for melanoma lung, kidney, and bladder cancer (3). Both CTLA-4 and PD-1 are expressed on T lymphocytes and negatively regulate the immune response. Canine lymphocytes also express PD-1 (26). The ligand for PD-1 on tumor cells is PD-L1 that generally is not expressed on normal cells. Studies using canine tumor biopsy samples and a human mAb that cross-reacts with canine PD-L1 confirm expression of PD-L1 on a number of canine tumors (24). However, a recent clinical trial with a “caninized” mAb against canine PD-L1 showed a (limited) response in dogs with advanced melanoma (27).

### Tumor-Specific Lymphocytes

Repeated infusions of autologous lymphocytes expanded on artificial antigen presenting cells stimulated with an anti-CD3 mAb and the (human) cytokines IL-2 and IL-21 improved overall survival in dogs with lymphoma post chemotherapy (28). However, this was not a controlled randomized study, and although the expansion protocol boosted the number of CD8-positive cytotoxic T cells, those were not tumor specific. The study confirmed that the human cytokines (IL-2, IL-15, and IL-21) can support the *ex vivo* expansion of canine T-cells, although higher doses are required.

Tumor-infiltrating lymphocytes (TILs), which are believed to be more specific toward cancer antigens, can be isolated from the tumor site and expanded *ex vivo* for treatment [reviewed in Ref. (29)]. The TIL approach requires not only substantial T-cell expansion but also more information about tumor-specific peptides in canine cancers. In addition, the peptide-presenting MHC complex is often mutated (or even missing) on cancer cells which impedes antigen presentation and T-lymphocyte interaction/activation. Considering the high costs of this intervention, it is clearly not of high priority for development as an immunotherapy for dogs.

Recently, CAR-engineered T-cells have made major news in human immunotherapy. The principle is to take the variable scFv region of a mAb and link it to sequences that can activate the cytotoxic T cell to recognize and kill the tumor cells (30). Essentially, this represents the antigen binding “tip” of a mAb engineered into the surface membrane of a T cell. CAR T-cells that target the CD19 receptor on human leukemia and lymphoma cells have resulted in some impressive responses, and the FDA just approved the first CAR T-cell treatment for patients with CD19-positive acute lymphoblastic leukemia (ALL).^1^ Although remissions could be achieved in over 50% of patients by day 28, late relapses continue to occur. CAR T-cell treatment can also cause significant toxicities such as cytokine release syndrome (CRS), encephalopathy or bone marrow suppression in about a third of treated patients (30). The logistics of generating CAR T cells and the substantial costs are certainly not very attractive for canine treatment; moreover, little is known about tumor-associated antigens as targets in canine cancer.

A team at MD Anderson Cancer Center transfected canine lymphocytes with a *human* HER-2 CAR and could show that the human CAR can target and kill HER-2-positive canine osteosarcoma cell lines (16). They could also show that HER-2-specific T lymphocytes from dogs can be successfully expanded. However, they had to use a feeder layer of human K562 cells engineered to express various human stimulatory antigens plus human IL-2 and IL-21. A similar CAR expansion protocol for the CD20 lymphoma antigen CD20 was recently presented (31). The investigators were able to treat one dog with those engineered cells, but only with partial tumor response. Both studies however illustrated the extensive process necessary to get CAR T-cells for dogs and its limitations making it rather unlikely that this treatment will become mainstream for canine cancer patients.

### NK Cells

Human NK cells are identified by the expression of CD56 and lacking CD3 and the T-cell receptor (TCR) (32). In contrast, canine NK cells do not express those “typical” NK cell surface markers. They lack the characteristic T-lymphocyte markers CD3 and express CD5^dim^ (33, 34). Recently, an antibody against the canine p46 receptor has been described that may help in NK cell isolation and characterization (35). Sources of cells for human NK cells are peripheral blood, placenta, or CD34-expressing progenitor cells from cord blood that can be expanded in culture into NK cells. The continuously growing human NK cell line NK-92 can provide an unlimited number of “off the shelf” NK cells and has recently been show to cross-react with canine cancer targets (36, 37). For dogs, only blood lymphocytes have been utilized as NK cell source. The group at UC Davis could shows that NK cells isolated from blood lymphocytes that were injected intra-tumor (osteosarcoma) can lead to tumor regression, an effect that can be augmented with local tumor radiation (38). In addition to providing “spontaneous” cytotoxicity (in contrast to T-cells, no priming is required), NK cells are important effector cells in mAb-mediated ADCC, a mechanism that in humans accounts for most of the anti-tumor effect of mAbs.

### Cytokines

Even for humans, cytokines by themselves have not made a major impact in immunotherapy. Interleukin-2 at higher doses can have significant side effects and can also induce unwanted T-regulatory immunosuppressive cells. Other immune-active cytokines, such as IL-12, IL-15, and IL-21, also have unacceptable toxicities [reviewed in Ref. (39)]. Although some human cytokines are effective in dogs, much higher doses are often needed and dogs can develop neutralizing antibodies against them (28, 40). Recently, the development of canine IL-15 has been reported (41). As a part of a Comparative Oncology Trial, *human* IL-12 was recently tested in a Phase I safety/dose escalation study in dogs with melanoma (42). Although the anti-tumor effect was limited, this study is a good example of how dogs can aid the design of studies in humans and guide drug development. It is hoped that those comparative oncology trials will also bring benefit for dogs with cancer and not just utilize the dog as a better mouse model.

Direct intra-tumor injection (i.e., via electrogen therapy) of plasmid DNA encoding the IL-12 cytokine has shown some benefit for some canine cancers (43, 44). However, the mechanism that triggers the anti-tumor response and whether the cytokine is immunologically active or merely induces a non-specific inflammatory response remains to be determined.

### Tumor Vaccines

Tumor vaccines are an appealing immunotherapy because the current methods of preparing the vaccines are usually less involved and their administration is relatively easy. Vaccines can be prepared from a number of sources. This has resulted in a plethora of tumor vaccines offered for dogs without necessarily showing proof of efficacy in proper controlled clinical trials.

In humans, treatment results with tumor vaccines have shown limited benefit if any at all, largely because the patient’s immune system is often compromised by the underlying malignancy and treatment. Furthermore, cancer cells are known to produce immunosuppressive molecules and to induce T-regulatory cells and macrophage-derived suppressor cells (MDSC) (45). It is believed that the injection of the vaccine can causes a localized inflammatory response resulting in non-specific immune activation which have led to the thinking that those vaccines should be combined with checkpoint inhibitors.

The first USDA-approved and commercially available tumor vaccine for canines is Oncept^®^, which targets the (human) tyrosinase protein in melanoma. This vaccine was greeted with enthusiasm, but investigators are still determining if the treatment has true therapeutic benefits beyond the peace of mind for dog owners in knowing that some intervention is being provided for their pet (46). It is beyond the scope of this review to discuss those various studies in detail only to suggest that a larger well-controlled study could put an end to the discussion. However, since the vaccine is approved and alternative treatment options are limited in melanoma, it is quite unlikely that this study will ever be done.

Studies on tumor vaccines and other therapies point to a general problem in testing new therapeutics in canines: Initial safety studies are done in a limited number of dogs and may show the occasional tumor response. However, follow-up trials in the more controlled clinical trials settings are infrequent. Consequently, we rarely know if initial positive, mostly anecdotal observations convey any true benefit to canine patients.

### Immune-Modulatory Treatments

A host of non-specific molecules, also called *biological response modifiers*, are being considered for the stimulation of innate canine immune effector cells such as NK cells, monocytes, and macrophages. For example, toll-like receptors (TLRs) are transmembrane glycoproteins that recognize molecules that are conserved among microbes (47). In *in vitro* studies, engagement of TLRs activates immune cells, resulting in upregulation of cytokines, chemokines, and costimulatory molecules, thereby supporting an adaptive immune response (48). Compared to murine studies, observations in humans and dogs have been less promising, suggesting possible differences in the cellular expression of TLRs in various species.

Many veterinarians will administer Cox-2 inhibitors to their cancer patients based on the experimental observation that local prostaglandins levels are increased which can suppress the immune response. This low cost treatment does not cause side effects and hence makes it an attractive immune intervention although controlled data to its efficacy are lacking.

Unfortunately, some companies are taking advantage of the less stringent regulations for nutraceuticals, vitamins, plant extracts, and similar treatments. Claims for many of these treatments include that they simulate the immune system. Although some plant-based compounds may affect *in vitro* cell-killing assays, scientifically sound proof of *in vivo* efficacy is lacking.

Finally, we should not forget that chemotherapy at low doses and even local tumor radiation can have positive effects on the immune system (49). Cytotoxic agents at a dose that does not necessarily kill tumor cells can induce stress ligands on cancer cells that are recognized by T and NK cells. Investigators have suggested to name this “pharmacological inducers of *immunogenic cell death (ICD)*.”

## Conclusion

Veterinarians, researchers, and dog owners hope that the strong interest in immunotherapy in the human oncology community will benefit canines as well. Requirements for success include better characterization of the canine immune system and its effector cells and molecules. One must also ensure that stringent outcome-assessment criteria are applied in studies of novel treatments. We will also have to accept that for canines, some immunotherapies will no doubt be challenging to prepare and to administer and will be too expensive to the extent that they may not become attractive candidate for pharmaceutical companies to develop for the veterinary market.

## Author Contributions

The author confirms being the sole contributor of this work and approved it for publication.

## Conflict of Interest Statement

The author is currently VP of Research and Development at NantKwest. He is also co-founder of and equity holder in NantKwest. The reviewer MP and handling Editor declared their shared affiliation.

## Footnotes

^1^https://www.cancer.gov/news-events/cancer-currents-blog/2017/tisagenlecleucel-fda-childhood-leukemia.
